# A Comparative Data-Based Modeling Study on Respiratory CO_2_ Gas Exchange during Mechanical Ventilation

**DOI:** 10.3389/fbioe.2016.00008

**Published:** 2016-02-03

**Authors:** Chang-Sei Kim, J. Mark Ansermino, Jin-Oh Hahn

**Affiliations:** ^1^Department of Mechanical Engineering, University of Maryland College Park, College Park, MD, USA; ^2^Department of Anesthesiology, Pharmacology and Therapeutics, The University of British Columbia, Vancouver, BC, Canada

**Keywords:** respiratory CO_2_ gas exchange, data-based modeling, closed-loop mechanical ventilation control

## Abstract

The goal of this study is to derive a minimally complex but credible model of respiratory CO_2_ gas exchange that may be used in systematic design and pilot testing of closed-loop end-tidal CO_2_ controllers in mechanical ventilation. We first derived a candidate model that captures the essential mechanisms involved in the respiratory CO_2_ gas exchange process. Then, we simplified the candidate model to derive two lower-order candidate models. We compared these candidate models for predictive capability and reliability using experimental data collected from 25 pediatric subjects undergoing dynamically varying mechanical ventilation during surgical procedures. A two-compartment model equipped with transport delay to account for CO_2_ delivery between the lungs and the tissues showed modest but statistically significant improvement in predictive capability over the same model without transport delay. Aggregating the lungs and the tissues into a single compartment further degraded the predictive fidelity of the model. In addition, the model equipped with transport delay demonstrated superior reliability to the one without transport delay. Further, the respiratory parameters derived from the model equipped with transport delay, but not the one without transport delay, were physiologically plausible. The results suggest that gas transport between the lungs and the tissues must be taken into account to accurately reproduce the respiratory CO_2_ gas exchange process under conditions of wide-ranging and dynamically varying mechanical ventilation conditions.

## Introduction

It is anticipated that autonomous closed-loop controlled mechanical ventilators will be increasingly used in the future to enhance the safety of mechanical ventilation. Automation will enable standardized treatment protocols as well as fill the gap between the increasing demands versus the limited number of respiratory experts. First, it is estimated that the failure to use recommended respiratory interventions in the intensive care unit (ICU) results in 170,000 preventable deaths per year in US (Pronovost et al., 2004). Thus, closed-loop-controlled mechanical ventilators can be an attractive option to translate the research knowledge into clinical practice *via* automatic knowledge transfer, which can potentially reduce errors, inappropriate interventions, and heterogeneity of knowledge and practice. Second, the number of prolonged mechanical ventilations (>96 h) was projected to have more than doubled in 2020 compared with that in 2000 (Zilberberg et al., 2008). It was also forecasted that the shortage of medical personnel with expertise in mechanical ventilation will begin in 2007 and will worsen thereafter (Angus et al., 2000). In this regard, autonomous and closed-loop-controlled mechanical ventilators can be a viable solution to guarantee quality care without exacerbating the medical personnel’s workload.

Despite their promising potential, closed-loop mechanical ventilation controllers have not yet penetrated, at least as much as anticipated, into clinical practice. This may be attributed to many reasons [such as conservatism and inertia (Cabana et al., 1999; Dent and Goldberg, 1999; Rubenfeld et al., 2004)], but a critical challenge may have been the concerns raised on the safety of these autonomous systems. Indeed, proving the validity and safety of closed-loop-controlled mechanical ventilators in preclinical testing is not trivial (see the limited number of subjects used in the testing of closed-loop mechanical ventilation controllers reported in the literature summarized in Table 1). In fact, even the new modes available in the commercialized mechanical ventilators [such as PAV (Younes, 2002), NAVA (Sinderby et al., 1999), ASV (Laubscher et al., 1994), and SmartCare™ (Dojat et al., 1992)] have not been extensively evaluated (Verbrugghe and Jorens, 2011; Cordioli et al., 2013; Rose et al., 2013).

**Table 1:** **Preclinical testing of closed-loop mechanical ventilation controllers reported in the literature**.

Reference	Endpoint	Controller	Model	Tuning	Testing
Ohlson et al. (1982)	$P_{{\text{etCO}}_{2}}$	PID	×	*Ad hoc*	6 (Dogs)
Ritchie et al. (1987)	$P_{{\text{etCO}}_{2}}$	PID	×	*Ad hoc*	5 (Dogs)
Laubscher et al. (1994)	RR and TV	PI	×	*Ad hoc*	6
Linton et al. (1994)	RR and TV	PI	×	*Ad hoc*	27
Schäublin et al. (1996)	$P_{{\text{etCO}}_{2}}$	Fuzzy logic	×	*Ad hoc*	30
Nemoto et al. (1999)	PSV level	Fuzzy logic	×	*Ad hoc*	13 (Retrospective)
Fernando et al. (2002)	MMV level	Alveolar ventilation equation	○	*Ad hoc*	1
Martinoni et al. (2004)	$P_{{\text{etCO}}_{2}}$	Observer feedback + PI	○	N/A	15
Jandre et al. (2004)	$P_{{\text{etCO}}_{2}}$	PI	×	*Ad hoc*	6 (Piglets)
Tehrani et al. (2004)	$P_{{\text{etCO}}_{2}}$	Empirical steady-state model	○	N/A	6 (Pigs)
	$F_{{\text{iO}}_{2}}$	PID + stepwise control			
Hahn et al. (2012)	$P_{{\text{etCO}}_{2}}$	PI	○	Root locus	18 (Models)

*In Model, “○” denotes model-based control, whereas “×” denotes non-model-based control. Tuning denotes how the controllers were tuned. Testing shows the subjects used to validate the controllers*.

*$P_{{\mathrm{etCO}}_{2}}$, end-tidal CO_2_ tension; RR, respiratory rate; TV, tidal volume; PSV, pressure support ventilation; MMV, mandatory minute ventilation; $F_{\text{i}O_{2}}$, inspiratory O_2_ tension*.

Mathematical models of physiologic system have been promoted as viable alternative to preclinical testing. Indeed, computational models may potentially be widely used to examine and assess the safety of a range of medical devices and systems, as suggested by the guidance document recently released by the U.S. Food and Drug Administration (FDA) on the reporting of computational modeling studies in the medical device submissions (CDRH and FDA, 2014). To rigorously assess the safety, efficacy and robustness of closed-loop mechanical ventilation controllers, a mathematical model of respiratory physiology is required that possesses several desired characteristics: (1) it must reproduce a realistic respiratory response to mechanical ventilation with accuracy for trustworthy assessment of mechanical ventilation controllers, (2) it must be low-order with minimum number of parameters for tractability in controller design and testing, and (3) it must be physiologically transparent to streamline the interpretation of the testing results. However, models of respiratory physiology reported in today’s literature do not fulfill all these characteristics. Most importantly, existing models are typically too complex to be used in Monte-Carlo simulation-based testing (Chiari et al., 1997; Anderson et al., 2003; Wolf and Garner, 2007; Cheng et al., 2010).

In pursuit of the ultimate goal of realizing a computational model of respiratory physiology applicable to the design and testing of closed-loop mechanical ventilation controllers, the goal of this study is to derive a minimally complex but credible model of respiratory CO_2_ gas exchange that may be used in systematic design and pilot testing of closed-loop end-tidal CO_2_ controllers in mechanical ventilation. We first derived a candidate model that captures the essential mechanisms involved in the respiratory CO_2_ gas exchange process. Then, we simplified the candidate model to derive two lower-order candidate models. We compared these candidate models for predictive capability and reliability using experimental data collected from 25 pediatric subjects undergoing dynamically varying mechanical ventilation during surgical procedures.

This paper is organized as follows. Section 2 outlines the model structure, its data-based modeling procedure, and the comparative analysis approaches. Section 3 describes the experimental data and data analysis methods. Section 4 presents and discusses the results. Section 5 concludes the paper with future directions.

## Respiratory Co_2_ Gas Exchange Model

### Model Structure Selection

**Figure 1:**
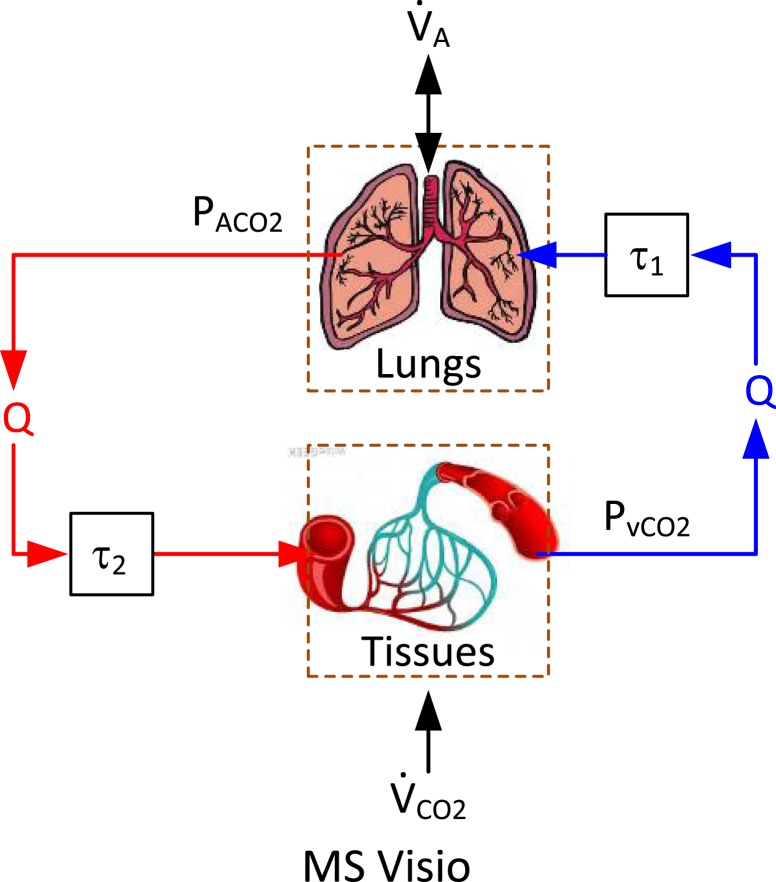
**Essential components in respiratory CO_2_ gas exchange process: the lungs, arteries and veins, and the body tissues**.

Respiratory CO_2_ gas exchange process during mechanical ventilation involves the lungs, arteries, and veins as well as body tissues (Grodins et al., 1954; Khoo et al., 1982; Batzel and Tran, 2000) (Figure 1). CO_2_ gas is produced in the tissues as a consequence of metabolism and is subsequently transported to the lungs *via* venous blood (whose CO_2_ tension is thus relatively high). CO_2_ gas is then excreted in the lungs by alveolar ventilation. The arterial blood, whose CO_2_ tension is relatively lower than the venous blood, is delivered back to the tissues to collect CO_2_ from them.

In this study, we model the lungs and the tissues as homogeneous compartments, while approximate the delivery of CO_2_ gas between them *via* arteries and veins as pure transport delays. The CO_2_ tension in the lungs (alveolar CO_2_ tension) is determined by the balance between CO_2_ supply from the venous blood and CO_2_ removal by the alveoli. The amount of CO_2_ gas supplied from the venous blood to the lungs depends on the difference between alveolar $\left(C_{{\text{ACO}}_{2}}\text{​}\right)$ versus mixed venous $\left(C_{{\text{vCO}}_{2}}\text{​}\right)$ CO_2_ concentrations as well as cardiac output (*Q*), while the amount of excreted CO_2_ gas is determined by alveolar CO_2_ tension $\left(P_{{\text{ACO}}_{2}}\text{​}\right)$ and alveolar ventilation $\left({\dot{V}}_{\text{A}}\right)$. Therefore, the rate of change in $P_{{\text{ACO}}_{2}}$ can be written as follows:

(1) $$V_{\text{L}}{\dot{P}}_{{\text{ACO}}_{2}}={\dot{V}}_{\text{A}}\left[P_{{\text{ICO}}_{2}}-P_{{\text{ACO}}_{2}}\right]+\lambda Q\left[C_{{\text{vCO}}_{2}}(t-\tau_{1})-C_{{\text{ACO}}_{2}}\right]$$

where *V*_L_ is the effective lung volume, $P_{{\text{ICO}}_{2}}$ is the CO_2_ tension in the inspired air, τ_1_ is the transport delay between the tissues and the lungs, and λ = 863 mmHg l_BTPS_/l_STPD_. The CO_2_ tension in the tissues $\left(C_{{\text{vCO}}_{2}}\right)$ is determined by the production of CO_2_ due to the body’s metabolic activity $\left({\dot{V}}_{{\text{CO}}_{2}}\right)$ and its removal from the tissues *via* arterial blood. The amount of CO_2_ gas removed from the tissues depends on the difference between $C_{{\text{ACO}}_{2}}$ versus $C_{{\text{vCO}}_{2}}$ as well as *Q*. Therefore, the rate of change in $C_{{\text{vCO}}_{2}}$ can be written as follows:

(2) $$V_{\text{B}}{\dot{C}}_{{\text{vCO}}_{2}}=Q\left[C_{{\text{ACO}}_{2}}\left(t-\tau_{2}\right)-C_{{\text{vCO}}_{2}}\right]+{\dot{V}}_{{\text{CO}}_{2}}$$

where *V*_B_ is effective tissue volume in the body and τ_2_ is the transport delay between the heart and the tissues. By assuming that CO_2_ concentration and tension are related to each other *via* the Henry’s law (Khoo et al., 1982; Lumb, 2005):

(3) $$C_{x{\text{CO}}_{2}}=\alpha P_{x{\text{CO}}_{2}}+\beta ,\;x=A,v$$

where α = 0.0065 l_STPD_/mmHg l, β = 0.244 l_STPD_/l, Eqs 1 and 2 are reduced to the following with $P_{{\text{ACO}}_{2}}$ and $P_{{\text{vCO}}_{2}}$ as state variables:

(4) $$\begin{array}{l} V_{\text{L}}{\dot{P}}_{{\text{ACO}}_{2}}={\dot{V}}_{\text{A}}\left[P_{{\text{ICO}}_{2}}-P_{{\text{ACO}}_{2}}\right]+\alpha \lambda Q\left[P_{{\text{vCO}}_{2}}\left(t-\tau_{1}\right)-P_{{\text{ACO}}_{2}}\right]\\ V_{\text{B}}{\dot{P}}_{{\text{vCO}}_{2}}=Q\left[P_{{\text{ACO}}_{2}}\left(t-\tau_{2}\right)-P_{{\text{vCO}}_{2}}\right]+\frac{{\dot{V}}_{{\text{CO}}_{2}}}{\alpha } \end{array}$$

Finally, denoting $x_{1}=P_{{\text{ACO}}_{2}}$, $x_{2}=P_{{\text{vCO}}_{2}}$, and $u={\dot{V}}_{\text{A}}$, and noting that $P_{{\text{ICO}}_{2}}=\text{0}$, yields the following state variable representation that dictates the respiratory gas exchange process during mechanical ventilation:

(5) $$\begin{array}{l} {\dot{x}}_{1}\left(t\right)=-\theta_{1}x_{1}\left(t\right)u\left(t\right)+\theta_{2}\left[x_{2}\left(t-\tau_{1}\right)-x_{1}\left(t\right)\right]\\ {\dot{x}}_{2}\left(t\right)=\theta_{3}\left[x_{1}\left(t-\tau_{2}\right)-x_{2}\left(t\right)\right]+\theta_{4} \end{array}$$

where $\theta_{1}=\frac{1}{V_{\text{L}}}$, $\theta_{2}=\frac{\alpha \lambda Q}{V_{\text{L}}}$, $\theta_{3}=\frac{Q}{V_{\text{B}}}$, and $\theta_{4}=\frac{{\dot{V}}_{{\text{CO}}_{2}}}{\alpha V_{\text{B}}}$ and the transport delays are the unknowns to be derived from the data. It is noted that *Q* and ${\dot{V}}_{{\text{CO}}_{2}}$ are regarded as constants (see Limitation and Future Perspectives for details).

We intend to conduct data-based modeling using minute ventilation $\left(\dot{V}\right)$ and end-tidal CO_2_ tension $\left(P_{{\text{etCO}}_{2}}\text{​}\right)$ data. To this aim, we assume that (1) $\dot{V}\approx {\dot{V}}_{\text{A}}$ (i.e., negligible dead space ventilation; see Limitation and Future Perspectives for details) and (2) $P_{{\text{etCO}}_{2}}\approx P_{{\text{ACO}}_{2}}$ (Burton, 1966; Coles et al., 1973; Ritchie et al., 1987; Williams and Babb, 1997; Brunner, 2002) (see Limitation and Future Perspectives for details). Since Eq. 5 cannot be identified solely based on $\dot{V}$ and $P_{{\text{etCO}}_{2}}$ because *x*_2_ is not accessible, it is re-formulated into the following regression on $\ddot{x}$_1_:

(6) $$\begin{array}{l} {\ddot{x}}_{1}\left(t\right)=-\theta_{1}\frac{d\left[x_{1}\left(t\right)u\left(t\right)\right]}{\mathrm{dt}}+\theta_{2}\left[{\dot{x}}_{2}\left(t-\tau_{1}\right)-{\dot{x}}_{1}\left(t\right)\right]\\ \;=\Omega_{3}^{T}\left[\begin{array}{c} -{\dot{x}}_{1}\left(t\right)u\left(t\right)-x_{1}\left(t\right)\dot{u}\left(t\right)\\ -{\dot{x}}_{1}\left(t\right)\\ -x_{1}\left(t\right)u\left(t\right)\\ x_{1}\left(t-\tau \right)-x_{1}\left(t\right)\\ 1 \end{array}\right] \end{array}$$

where $x_{1}=P_{{\text{etCO}}_{2}}$, $u=\dot{V}$, τ = τ_1_ + τ_2_, and $\Omega_{3}={\left[\begin{array}{ccc} \begin{array}{cc} \theta_{1} & \theta_{2} \end{array}+\theta_{3} & \begin{array}{c} \theta_{1}\theta_{3} \end{array} & \begin{array}{cc} \theta_{2}\theta_{3} & \theta_{2}\theta_{4} \end{array} \end{array}\right]}^{\text{T}}$. Note that only τ, but not τ_1_ and τ_2_ individually, appears in Eq. 6 because only *x*_1_ is measured. Both $P_{{\text{etCO}}_{2}}$ and $\dot{V}$ data are given by discrete-time sequences. Thus, the regression Eq. 6 is discretized using the forward difference approximation (Nakamura, 1993) (this model is called M3 hereafter):

(7) $$\begin{array}{l} {\ddot{x}}_{1}\left(k\right)=\frac{x_{1}\left(k+2\right)-2x_{1}\left(k+1\right)+x_{1}\left(k\right)}{T_{s}^{2}}\\ \;=\Omega_{3}^{T}\left[\begin{array}{c} -\frac{x_{1}\left(k+1\right)-x_{1}\left(k\right)}{T_{s}}u\left(k\right)-x_{1}\left(k\right)\frac{u\left(k+1\right)-u\left(k\right)}{T_{s}}\\ -\frac{x_{1}\left(k+1\right)-x_{1}\left(k\right)}{T_{s}}\\ -x_{1}\left(k\right)u\left(k\right)\\ x_{1}\left(k-\tau \right)-x_{1}\left(k\right)\\ 1 \end{array}\right]\\ \;=\Omega_{3}^{T}\psi_{3}\left(k,\tau \right) \end{array}$$

where *T*_s_ is sampling interval and τ is restricted to positive integers to be compatible with the discretization. Since the regression vector in Eq. 7 can be constructed solely based on *x*_1_ and *u*, $\Theta ≜\left\{\theta_{1},\theta_{2},\theta_{3},\theta_{4}\right\}$ and τ may be identified by fitting Eq. 6 to $P_{{\text{etCO}}_{2}}$ and $\dot{V}$.

We considered two avenues in deriving lower-order models from Eq. 4. First, noting that transport delays were not taken into account in many previous studies [see, e.g., Melo et al. (1993), Olofsen et al. (2010), Wang et al. (2010), and Karbing et al. (2011) versus Grodins et al. (1967), Khoo et al. (1982), Batzel and Tran (2000), Beda et al. (2010), and Dunn and Whiteley (2010)], a lower-order model was derived by neglecting τ in Eq. 4. This results in the following regression model (called M2 hereafter):

(8) $$\begin{array}{l} {\ddot{x}}_{1}\left(k\right)=\frac{x_{1}\left(k+2\right)-2x_{1}\left(k+1\right)+x_{1}\left(k\right)}{T_{s}^{2}}\\ \;=\Omega_{2}^{T}\left[\begin{array}{c} -\frac{x_{1}\left(k+1\right)-x_{1}\left(k\right)}{T_{s}}u\left(k\right)-x_{1}\left(k\right)\frac{u\left(k+1\right)-u\left(k\right)}{T_{s}}\\ -\frac{x_{1}\left(k+1\right)-x_{1}\left(k\right)}{T_{s}}\\ -x_{1}\left(k\right)u\left(k\right)\\ 1 \end{array}\right]\\ \;=\Omega_{2}^{T}\psi_{2}\left(k\right) \end{array}$$

where $\Omega_{2}={\left[\begin{array}{cc} \begin{array}{cc} \theta_{1} & \theta_{2}+\theta_{3} \end{array} & \begin{array}{cc} \theta_{1}\theta_{3} & \theta_{2}\theta_{4} \end{array} \end{array}\right]}^{\text{T}}$. Second, Eq. 8 can be further simplified by aggregating the compartments associated with the lungs and the tissues. If we assume that gas exchange and metabolism occur in a single compartment representing the lungs and the tissues altogether, the following regression model results from Eq. 4 by setting *x*_1_ = *x*_2_ and τ_1_ = τ_2_ = 0, and then combining the two equations in Eq. 4 (called M1 hereafter):

(9) $${\dot{x}}_{1}\left(k\right)=\frac{x_{1}\left(k+1\right)-x_{1}\left(k\right)}{T_{s}}=\Omega_{1}^{T}\left[\begin{array}{c} -x_{1}\left(k\right)u\left(k\right)\\ 1 \end{array}\right]=\Omega_{1}^{T}\psi_{1}\left(k\right)$$

where $\Omega_{1}={\left[\begin{array}{c} \begin{array}{cc} \frac{1}{V_{\text{L}}+\alpha \lambda V_{\text{B}}} & \frac{\lambda {\dot{V}}_{{\text{CO}}_{2}}}{V_{\text{L}}+\alpha \lambda V_{\text{B}}} \end{array} \end{array}\right]}^{\text{T}}$.

### Model Identification

Since Eq. 7 is a pseudo-linear regression model due to the dependence of the regression vector ψ_3_ on τ, it cannot be solved *via* standard linear least-squares method. In addition, its parameter vector Ω_3_ contains the products of unknowns θ_1_θ_3_, θ_2_θ_3_, and θ_2_θ_4_, which presents a challenge in deriving θ*_*i*_*, *i* = 1, …, 4 from Ω_3_ uniquely. Further, standard least-squares method is not ideal in maximizing the predictive capability since it minimizes one-step-ahead prediction error instead of pure (i.e., infinite-step-ahead) prediction error. To cope with these challenges, we identified Θ and τ by solving the following optimization problem:

(10) $$\left\{\Theta^{\text{*}},\tau^{\text{*}}\right\}=\mathrm{arg min J}=\mathrm{arg min}\frac{1}{N}\sum_{k=1}^{N}{\left[x_{1}\left(k\right)-{\hat{x}}_{1}\left(k|\Theta ,\tau \right)\right]}^{2}$$

where {Θ^*^,τ^*^} is the set of optimal model parameters, *N* is the number of data samples used to solve Eq. 10, *x*_1_(*k*) is the $P_{{\text{etCO}}_{2}}$ data at sample time *k*, while ${\hat{x}}_{1}\left(k|\Theta ,\tau \right)$ is the model-predicted $P_{{\text{etCO}}_{2}}$ derived from Eq. 7 by

(11) $${\hat{x}}_{1}\left(k|\Theta ,\tau \right)=2{\hat{x}}_{1}\left(k-1\right)-{\hat{x}}_{1}\left(k-2\right)+T_{\text{s}}^{2}\Omega_{3}^{\text{T}}{\hat{\psi }}_{3}\left(k-2,\tau \right)$$

where ${\hat{\psi }}_{3}\left(k,\tau \right)$ is ψ_3_ computed using ${\hat{x}}_{1}\left(k\right)$’s, *u*(*k*)’s, and Ω_3_ = Ω_3_(Θ). Once Θ is determined, the physical respiratory parameters can be derived as follows. First, *V*_L_ is derived as $V_{\text{L}}=\theta_{1}^{-1}$. Second, *Q* is derived as $Q=\frac{V_{\text{L}}}{\alpha \lambda }\theta_{2}$. Third, *V*_B_ is derived as $V_{\text{B}}=\frac{Q}{\theta_{3}}$. Finally, ${\dot{V}}_{{\text{CO}}_{2}}$ is derived as ${\dot{V}}_{{\text{CO}}_{2}}=\alpha V_{\text{B}}\theta_{4}$. Thus, *V*_L_, *V*_B_, *Q*, and ${\dot{V}}_{{\text{CO}}_{2}}$ can be uniquely derived from Θ.

The parameters in M1 and M2 can be determined by solving optimization problems similar to Eq. 10 in order to maximize predictive capability, so that an objective comparison can be made on the three candidate models.

### Comparative Model Analysis

The three candidate models were compared in terms of predictive capability and reliability. First, the predictive capability was measured in terms of the root-mean-squared error (RMSE) between $P_{{\text{etCO}}_{2}}$ data [*x*_1_(*k*)] versus model-predicted $P_{{\text{etCO}}_{2}}$ (${\hat{x}}_{1}\left(k|\Theta^{\text{*}},\tau^{\text{*}}\right)$; note that τ^*^ = 0 for M1 and M2):

(12) $$\text{RMSE}=\sqrt{\frac{1}{N}\sum_{k=1}^{N}\varepsilon^{2}\left(k\right)}=\sqrt{\frac{1}{N}\sum_{k=1}^{N}{\left[x_{1}\left(k\right)-{\hat{x}}_{1}\left(k|\Theta^{\text{*}},\tau^{\text{*}}\right)\right]}^{2}}$$

Second, the reliability was measured in terms of the asymptotic variance (Ljung, 1999), which represents the expected parametric variance estimated by the prediction error and the parametric sensitivity:

(13) $$\begin{array}{l} \text{Var}\left[\Theta_{0}-\Theta^{\text{*}}\right]\approx \frac{1}{N}\lambda_{N}\left(\Theta^{\text{*}}\right){\text{S}}_{N}\left(\Theta^{\text{*}}\right)\\ \;=\frac{1}{N}\lambda_{N}\left(\Theta^{\text{*}}\right){\left[\frac{1}{N}\sum_{k=1}^{N}\frac{\text{d}\ddot{\varepsilon }\left(k\right)}{\text{d}\Theta }\frac{\text{d}{\ddot{\varepsilon }}^{\text{T}}\left(k\right)}{\text{d}\Theta }\right]}^{-1} \end{array}$$

where $\lambda_{N}\left(\Theta^{\text{*}}\right)=\frac{1}{N}\sum_{k=1}^{N}{\ddot{\varepsilon }}^{2}\left(k\right)$ is the prediction error variance associated with $\ddot{\varepsilon }\left(k\right)$, and *S_*N*_*(Θ^*^) is the inverse sensitivity covariance matrix. Finally, the Akaike’s Information Criterion (AIC) (Burnham and Anderson, 1998) was used to compare the overall quality of the candidate models by rewarding the predictive capability and penalizing the model complexity simultaneously:

(14) $$\text{AIC}\approx N\ln\left[\frac{1}{N}\sum_{k=1}^{N}{\ddot{\varepsilon }}^{2}\left(k\right)\right]+2K+\frac{2K\left(K+1\right)}{N-K-1}$$

where *K* is the number of parameters in the model. The first term in Eq. 14 denotes the predictive capability (i.e., the goodness of fit), while the second term reflects the model complexity. The third term is to compensate for finite sample size (Burnham and Anderson, 1998).

## Methods

### Experimental Data

The $P_{{\text{etCO}}_{2}}$ and $\dot{V}$ data used in this study were extracted from a large and anonymized physiologic database, which was constructed out of a standardized set of clinical data collected as part of a larger investigation which was approved by the Children’s and Women’s Health Centre of British Columbia. We selected 25 anonymous pediatric subjects receiving pressure-controlled mechanical ventilation during a surgical procedure. These subjects were selected based on the evidence of a large change in the ventilation setting made by the caregivers, manifested by their variability in respiratory rate (RR), peak inspiratory pressure, and I:E ratio (the ratio between inspiratory time versus expiratory time), rendering the corresponding data appropriate for data-based modeling analysis. The data were collected as trend values every 5 s using a standard respiratory module (M-CAiOVX, Datex-Ohmeda, Finland) and then saved *via* a custom-built, centrally located data collection software.

### Data Analysis

The $P_{{\text{etCO}}_{2}}$ and $\dot{V}$ data thus recorded were used to solve the optimization problems (e.g., Eq. 10 for M3) to derive the candidate models for each subject. A constrained optimization routine in MATLAB’s Optimization Toolbox was used. Multiple initial parameter values were employed to assure that the solution converged to the global minimum. The model-predicted $P_{{\text{etCO}}_{2}}$ was produced by applying $u=\dot{V}$ and the requisite initial conditions on $x_{1}=P_{{\text{etCO}}_{2}}$ to Eq. 11. In deriving M3, the optimization problem was repetitively solved while the value of τ was varied *via* exhaustive search within a domain that was specified *a priori* based on the physiologically relevant values reported in the literature [see, e.g., Khoo et al. (1982) and Batzel and Tran (2000)]. In this way, multiple candidate solution sets (i.e., {Θ^*^,τ^*^}) were derived for different τ. Then, the optimal solution set was determined as the one associated with the minimum *J* value.

Once the optimal data-based models (M1, M2, and M3) corresponding to the 25 subjects were derived and the corresponding model-predicted $P_{{\text{etCO}}_{2}}$ produced, the candidate models associated with each subject were compared with each other using RMSE, asymptotic variance and AIC, which were computed by Eqs 12–14, respectively. The number of data samples that was used to compute RMSE, asymptotic variance, and AIC was dependent on subjects, since the amount of data available from each subject was different (see Table 2). The significance in difference among the candidate models was assessed as follows. First, the difference in predictive capability was analyzed using the paired *t*-test. Second, the difference in reliability associated with each element in $\Theta^{\text{*}}≜\left\{\theta_{1}^{\text{*}},\theta_{2}^{\text{*}},\theta_{3}^{\text{*}},\theta_{4}^{\text{*}}\right\}$ was analyzed by applying the paired *t*-test to the asymptotic variance corresponding to each element in Θ^*^. The difference was regarded as significant in case *p* < 0.05. Third, in comparing the candidate models by AIC, the frequency in which M1, M2, and M3 attained the minimum AIC values was counted across the 25 subjects in order to assess how many times each of the candidate models was suggested as the best model among them.

**Table 2:** **The range of $P_{{\text{etCO}}_{2}}$, $\dot{V}$, mechanical ventilation settings, and the data length, shown in terms of median [interquartile range (IQR)]**.

	$P_{{\text{etCO}}_{2}}$ (mmHg)	$\dot{V}$ (lpm)	TV (ml)	RR (m^−1^)	*P*_peak_ (cmH_2_O)	I:E ratio	Length (min)
Median (IQR)	40.8 (38.2–43.6)	2.24 (1.84–2.59)	226 (197–250)	10.7 (9.4–12.3)	13.1 (12.5–13.8)	1.02 (0.79–1.23)	24.4 (12.3–36.6)
$\frac{\left(\text{Max}-\text{Min}\right)}{\text{Mean}}$	35.8%	89.1%	62.0%	60.9%	45.7%	108.8%	–

*$P_{{\mathrm{etCO}}_{2}}$, end-tidal CO_2_ tension; $\dot{V}$, minute ventilation; TV, tidal volume; RR, respiratory rate; *P*_peak_, peak inspiratory pressure*.

## Results and Discussion

A parsimonious and credible model of respiratory mechanics and physiology may expedite the design, analysis, testing, and deployment of closed-loop mechanical ventilation controllers. As an initial step toward such a computational model, the goal of this study was to derive a minimally complex and credible respiratory CO_2_ gas exchange model applicable to the design and pilot testing of closed-loop end-tidal CO_2_ controllers during mechanical ventilation. The novelty of this study is that we have rigorously compared respiratory CO_2_ gas exchange models with different degrees of complexity. We have evaluated the reliability and physiologic transparency of the respiratory parameters as well as the model accuracy in reproducing the relationship between minute ventilation and end-tidal CO_2_, which may offer unique value to the data-based computational modeling of respiratory physiology.

### Data

**Figure 2:**
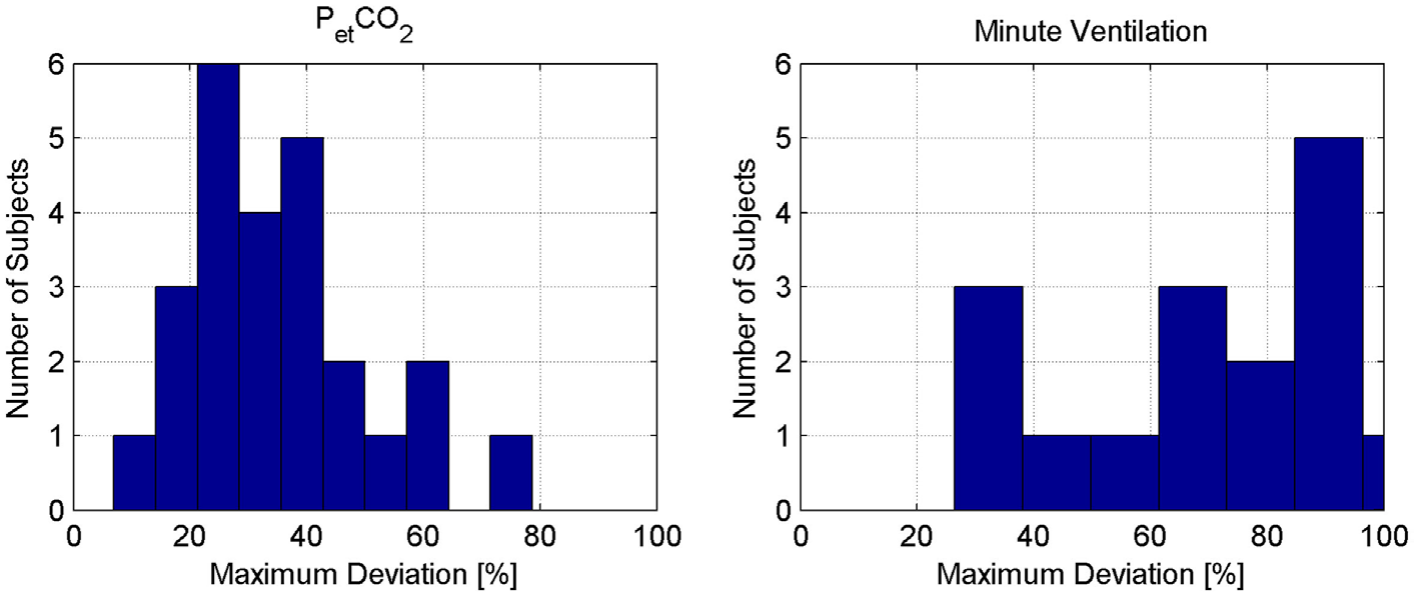
**Distribution of the maximum percentage deviation of $P_{{\text{etCO}}_{2}}$ and $\dot{V}$ from their respective within-subject mean values**.

On the average, 24.4 min length of data was available from each subject (Table 2). The ventilation settings were largely altered to yield considerable changes in $P_{{\text{etCO}}_{2}}$ and $\dot{V}$, as evidenced by Table 2 and Figure 2. In particular, Figure 2 shows that more than 30% change in $\dot{V}$ (from its within-subject mean value) was made in 24 subjects, resulting in more than 29% change in $P_{{\text{etCO}}_{2}}$ in those subjects (again, from its within-subject mean value).

### Predictive Capability and Reliability

**Figure 3:**
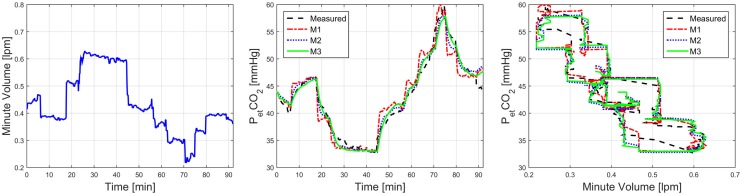
**Representative model-predicted $P_{{\text{etCO}}_{2}}$ in response to $\dot{V}$ associated with M1, M2, and M3**.

Overall, the two-compartment models (M2 and M3) exhibited superior predictive accuracy than their one-compartment counterpart (M1). Compared with M1, M2, and M3 achieved 35 and 45% reductions in RMSE, respectively, which were statistically significant (Table 3). A representative model-predicted $P_{{\text{etCO}}_{2}}$ in response to $\dot{V}$ is shown in Figure 3. In this subject, both RR (7–13 1/min) and tidal volume (TV; 31.2–56.9 ml/min) were varied to yield a large change in $\dot{V}$ and $P_{{\text{etCO}}_{2}}$. This example indicates that M2 and M3 can reproduce the relationship between $P_{{\text{etCO}}_{2}}$ and $\dot{V}$ with high fidelity, whereas M1 clearly exhibits deficiency. Due to its largely degraded predictive capability compared with M2 and M3, the remaining investigation was devoted to the comparison between M2 and M3.

**Table 3:** **RMSE, asymptotic variance, and AIC associated with the candidate models**.

	RMSE (mmHg)	Asymptotic variance				AIC
		$\theta_{1}^{\text{*}}$ (%)	$\theta_{2}^{\text{*}}$ (%)	$\theta_{3}^{\text{*}}$ (%)	$\theta_{4}^{\text{*}}$ (%)	
M1	1.95 (1.31–2.54)	–	–	–	–	3
M2	1.26 (0.88–1.80)	0.04 (0.01–0.30)	0.00 (0.00–0.00)	0.00 (0.00–0.00)	11.82 (1.5–118.6)	5
M3	1.08 (0.68–1.58)	0.21 (0.08–0.45)	0.01 (0.00–0.03)	0.36 (0.21–2.85)	3.90 (1.4–36.0)	17

*RMSE and asymptotic variance are shown in terms of median (IQR)*.

*AIC corresponding to each model denotes the number of subjects in which the model was selected as the best model*.

The asymptotic variance (expressed as asymptotic standard variation) associated with $\theta_{1}^{\text{*}}$, $\theta_{2}^{\text{*}}$, and $\theta_{3}^{\text{*}}$ was reasonably small in both M2 and M3, which suggests that these parameters were adequately identified. Considering that $\theta_{1}^{\text{*}}$, $\theta_{2}^{\text{*}}$, and $\theta_{3}^{\text{*}}$ are made up of ${\text{V}}_{\text{L}}^{\text{*}}$, ${\text{V}}_{\text{B}}^{\text{*}}$, and Q^*^ (which are closely related to the time constants associated with *x*_1_ and *x*_2_), reliable determination of these parameters may be attributed to the dynamic, widely varying $\dot{V}$ that resulted in informative transient components in the $P_{{\text{etCO}}_{2}}$ response. On the other hand, the asymptotic variance associated with $\theta_{4}^{\text{*}}$ was relatively large for both M2 and M3. This may be at least in part attributed to the underlying assumption in these models that ${\dot{V}}_{{\text{CO}}_{2}}^{\text{*}}$ is constant. Indeed, considering that ${\dot{V}}_{{\text{CO}}_{2}}^{\text{*}}=\alpha V_{\text{B}}^{\text{*}}\theta_{4}^{\text{*}}$ where $V_{\text{B}}^{\text{*}}$ can be reliably derived from $\theta_{2}^{\text{*}}$ and $\theta_{3}^{\text{*}}$, uncertainty in $\theta_{4}^{\text{*}}$ may arise from ${\dot{V}}_{{\text{CO}}_{2}}^{\text{*}}$. This interpretation is in fact supported by the examination of S_N_(Θ^*^) in Eq. 13. For all the 25 subjects, we found that the smallest eigenvalue of the sensitivity covariance matrix $S_{N}^{-1}\left(\Theta^{\text{*}}\right)$ associated with M2 and M3 was consistently aligned with the direction of $\theta_{4}^{\text{*}}$, which means that $P_{{\text{etCO}}_{2}}$ is more sensitive to $\theta_{1}^{\text{*}}$, $\theta_{2}^{\text{*}}$, and $\theta_{3}^{\text{*}}$ than $\theta_{4}^{\text{*}}$. The reason for the small sensitivity of $P_{{\text{etCO}}_{2}}$ to $\theta_{4}^{\text{*}}$ may be because the corresponding regressor element is constant (i.e., 1), whereas the rest of the elements in ψ_2_ and ψ_3_ are widely varying. It may thus be concluded that the relatively large asymptotic variance associated with $\theta_{4}^{\text{*}}$ is caused by the lack of excitation in its direction by the data, which is essentially due to the assumption that ${\dot{V}}_{{\text{CO}}_{2}}^{\text{*}}$ is constant.

The analysis of AIC suggested that M3 is overall superior to M1 and M2, while M2 is superior to M1. The AIC results indicate that transport delay plays a crucial role in reproducing the respiratory gas exchange process under dynamically varying mechanical ventilation conditions.

### Physiologic Transparency

The respiratory parameters derived for M2 were largely different from those for M3 with statistical significance (see Table 4). Compared with the respiratory parameter values for adults reported in the literature, the respiratory parameters derived for M3 appeared to be more physiologically plausible than those associated with M2. In particular, the median values of $V_{\text{L}}^{\text{*}}$, $V_{\text{B}}^{\text{*}}$, Q^*^, and ${\dot{V}}_{{\text{CO}}_{2}}^{\text{*}}$ derived for M3 were approximately 72, 31, 22, and 46% of the typical adult values, which appears to be at least qualitatively reasonable considering that the data used in this study were acquired from pediatric subjects.^1^ In contrast, the respiratory parameter values derived for M2 were unacceptably small to be physiologically realistic. Further investigation of the models showed that the predictive accuracy of M3 drastically deteriorated when its transport delay was set to 0 (much worse than M2), which suggests that the parameters derived for M2 were actually optimized reasonably well to minimize the prediction error in the absence of transport delay. Therefore, the above observation is an additional evidence to support that wide-ranging changes in mechanical ventilation settings result in emphasized transient responses in $P_{{\text{etCO}}_{2}}$ that may not be captured without transport delay.

**Table 4:** **Parameters identified from the data-based modeling analysis [median (IQR)]**.

	$V_{\text{L}}^{\text{*}}$ (l)	$V_{\text{B}}^{\text{*}}$ (l)	Q* (lpm)	${\dot{V}}_{{\text{CO}}_{2}}^{\text{*}}$ (l_STPD_/min)	τ* (s)
M1	–	–	–	–	–
M2	0.05 (0.03–0.11)	0.11 (0.04–0.31)	0.06 (0.04–0.13)	1 × 10^−4^ (1 × 10^−4^–3 × 10^−4^)	–
M3	2.28 (1.36–4.63)	4.67 (1.56–9.73)	1.20 (0.39–2.95)	0.12 (0.04–0.15)	20 (5–44)

The range of the respiratory parameters derived for M3 were wide; the interquartile ranges (IQRs) of $V_{\text{L}}^{\text{*}}$, $V_{\text{B}}^{\text{*}}$, Q^*^, and ${\dot{V}}_{{\text{CO}}_{2}}^{\text{*}}$ were approximately 143, 175, 213, and 92% of the respective median values. The results suggest that the subjects were associated with a wide-ranging respiratory variability, though the validity of the absolute values of the respiratory parameters cannot be established. The fact that our model could reproduce the responses of all these subjects without any *a priori* knowledge of these subjects is notable, because its governing Eq. 5 was derived by explicitly considering physiologic principles rather than to simply fit the data. In sum, the ability to derive subject-specific respiratory parameters suited to each subject is the strength of our model.

### Limitation and Future Perspectives

This study has a number of limitations that need to be addressed in follow-up studies.

First, the lungs of the subjects analyzed in this study were mostly normal without any lung disease. Therefore, the validity of our model in subjects with respiratory pathophysiology has yet to be evaluated. It may be that our model needs to be expanded to include more physiologic mechanisms relevant to accurately reproduce the CO_2_ gas exchange phenomena in the presence of lung diseases. Future work must make improvements to our model to develop and validate minimally complex lumped-parameter models of respiratory CO_2_ gas exchange that can be used for both normal and pathologic conditions.

Second, the subjects examined in this study were subject to different anesthesia protocols and surgical procedures. This may not alter the conclusion regarding the validity of our model, because the model could yield physiologically plausible respiratory parameter values that accurately reproduced the data in all the subjects examined in this study. However, the values of the respiratory parameters associated with each subject may have been modestly affected in different ways to capture the influence of different anesthesia protocols and surgical procedures. To solidly establish the validity of our model, it must be tested against data collected under strictly standardized protocols.

Third, several key mechanisms in the model were simplified. For example, CO_2_ production and cardiac output were assumed constant in the model presented in this study. Noting that the subjects were undergoing surgical procedures, it may be reasonable to assume that CO_2_ production and cardiac output were stable due to the effect of anesthesia and the end-tidal CO_2_ was varied mainly by the change in the mechanical ventilator settings. However, it is possible that CO_2_ production and cardiac output may have changed, which may in turn have had an influence on the model parameters. The validity of the model presented in this study may be limited in subjects undergoing a large change in CO_2_ production and/or cardiac output. The models in this study also assumed negligible dead space ventilation and pulmonary shunt because accurate subject-specific estimation of dead space ventilation and pulmonary shunt solely based on minute ventilation and end-tidal CO_2_ data is in general challenging. Though the subjects examined in this study were all healthy, even healthy subjects have non-zero dead space, which may further increase under anesthesia despite the mechanical ventilation. Thus, this assumption may have had an influence on the values of the model parameters. In addition, this assumption may be far less justified in subjects with lung disease, limiting the applicability of our model. Thus, our model must be improved to explicitly incorporate dead space ventilation and pulmonary shunt.

Fourth, the models presented in this study are continuous ventilation models. As such, the tidal nature of breathing was not explicitly incorporated in the models. It has been suggested that tidal ventilation models explicitly incorporating the discontinuity in breathing due to inspiratory and expiratory phases are more desirable than continuous ventilation models when attempting to obtain a rigorous and comprehensive understanding of physiology and pathophysiology in respiratory gas exchange during mechanical ventilation [see, e.g., Hahn and Farmery (2003)] and the references therein]. The model presented in this work was able to reproduce the end-tidal CO_2_ response to minute ventilation. On the other hand, the validity and utility of this model beyond end-tidal CO_2_ (e.g., the relationship between *x*_2_ and mixed venous CO_2_ concentration) still need to be investigated. In this regard, care must be taken in using this model in analyzing responses to mechanical ventilation other than end-tidal CO_2_. At the same time, efforts to extend our continuous ventilation model to include tidal nature of breathing may also be rewarding.

Finally, the model presented in this study did not incorporate oxygen (O_2_) gas exchange. Considering that the primary objective of mechanical ventilation therapy is to achieve adequate arterial oxygenation, a truly viable computational model of respiratory physiology for mechanical ventilation beyond closed-loop end-tidal CO_2_ control must incorporate O_2_ dynamics. Future work must be conducted to expand our CO_2_ gas exchange model to enable the reproduction of both O_2_ and CO_2_ responses to mechanical ventilation.

## Conclusion and Future Work

In our effort to derive a minimally complex, high-fidelity, and transparent data-based respiratory physiology model applicable to the design and pilot testing of closed-loop end-tidal CO_2_ controllers, we conducted a comparative data-based modeling analysis of the respiratory CO_2_ gas exchange process. It was shown that a two-compartment model with transport delay was able to accurately reproduce the end-tidal CO_2_ tension response to dynamically varying minute ventilation challenge in human subjects. Future work will include further investigation of the model as well as the design of model-based closed-loop end-tidal CO_2_ controllers.

## Author Contributions

All authors listed have made substantial, direct, and intellectual contribution to the work and approved it for publication.

## Conflict of Interest Statement

The authors declare that the research was conducted in the absence of any commercial or financial relationships that could be construed as a potential conflict of interest.

## Footnotes

^1^The weights of the pediatric subjects were not available in the anonymized data. However, the median size of the subjects may be estimated by comparing the values of $\dot{V}$ and TV in Table 2 with those of typical adult values. For example, the median $\dot{V}$ of 2.24 lpm is approximately 36.3% of the typical adult value of 6.16 lpm (TV 8.0 ml kg × weight 70 kg × RR 11 m), which is compatible to 72, 31, 22, and 46% of the typical adult values associated with $V_{L}^{*}$, $V_{B}^{*}$, Q, and ${\dot{V}}_{{\mathrm{CO}}_{2}}^{*}$, respectively.
